# Comprehensive Design of the High-Sulfur-Loading Li–S Battery Based on MXene Nanosheets

**DOI:** 10.1007/s40820-020-00449-7

**Published:** 2020-05-20

**Authors:** Shouzheng Zhang, Ning Zhong, Xing Zhou, Mingjie Zhang, Xiangping Huang, Xuelin Yang, Ruijin Meng, Xiao Liang

**Affiliations:** 1 College of Materials and Chemical Engineering, China Three Gorges University, 8 Daxue Road, Yichang, 443002 Hubei People’s Republic of China; 2 State Key Laboratory of Chem/Biosensing and Chemometrics, College of Chemistry and Chemical Engineering, Hunan University, Changsha, 410082 People’s Republic of China; 3 College of Science, China Three Gorges University, 8 Daxue Road, Yichang, 443002 Hubei People’s Republic of China

**Keywords:** MXene nanosheet, High sulfur areal loading, Interlayer, Lithium–sulfur battery

## Abstract

The lithium-sulfur battery is the subject of much recent attention due to the high theoretical energy density, but practical applications are challenged by fast decay owing to polysulfide shuttle and electrode architecture degradation. A comprehensive study of the sulfur host microstructure design and the cell architecture construction based on the MXene phase (Ti_3_C_2_T_*x*_ nanosheets) is performed, aiming at realize stable cycling performance of Li–S battery with high sulfur areal loading. The interwoven KB@Ti_3_C_2_T_*x*_ composite formed by self-assembly of MXene and Ktejen black, not only provides superior conductivity and maintains the electrode integrality bearing the volume expansion/shrinkage when used as the sulfur host, but also functions as an interlayer on separator to further retard the polysulfide cross-diffusion that possibly escaped from the cathode. The KB@Ti_3_C_2_T_*x*_ interlayer is only 0.28 mg cm^−2^ in areal loading and 3 μm in thickness, which accounts a little contribution to the thick sulfur electrode; thus, the impacts on the energy density is minimal. By coupling the robust KB@Ti_3_C_2_T_*x*_ cathode and the effective KB@Ti_3_C_2_T_*x*_ modified separator, a stable Li–S battery with high sulfur areal loading (5.6 mg cm^−2^) and high areal capacity (6.4 mAh cm^−2^) at relatively lean electrolyte is achieved.

**Electronic supplementary material:**

The online version of this article (10.1007/s40820-020-00449-7) contains supplementary material, which is available to authorized users.

## Highlights

- The intrinsic negatively charged MXene nanosheets were assembled to the positively charged Ketjen black/sulfur (KB/S) or KB to constructing the interwoven composite.
- The KB/S@Ti_3_C_2_T_x_ architectures allow high sulfur loading and accommodate the corresponding volume change, while the structural integrity and the ionic and electric conducting pathways are well maintained.
- The KB@Ti_3_C_2_T_x_ interlayers further retard the polysulfide that escaped from the cathode. It is only 0.28 mg cm^−2^ in density and 3 μm in thickness—the effect on energy density is minimal.

**Electronic supplementary material:**

The online version of this article (10.1007/s40820-020-00449-7) contains supplementary material, which is available to authorized users.

## Graphical abstract

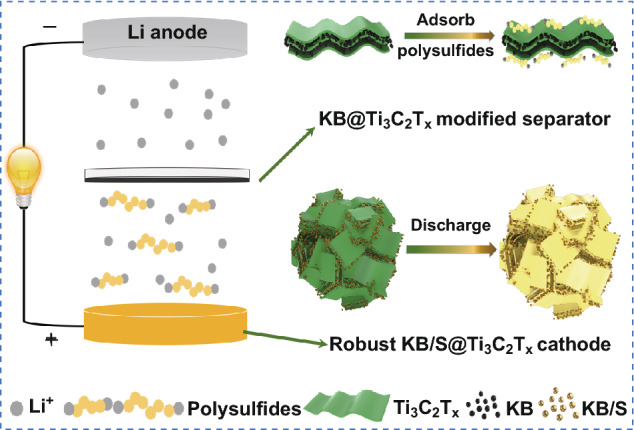

## Introduction

The ever-increasing demands from portable electronic devices, electric vehicles and renewable energy sources call for energy storage devices that embrace lower cost and higher energy density as well as longer cycling life [1]. Li–S batteries that couple earth-abundant and high-capacity sulfur positive electrodes with lithium negative electrodes are considered among the most promising candidates due to high theoretical specific capacity (1675 mAh g^−1^) and high theoretical energy density (2600 Wh kg^−1^) [2]. Nonetheless, obstacles that impede the application of Li–S batteries are fast capacity decay, poor rate capability and low practical energy density. The polysulfide shuttle, low conductive (electric and ionic) of sulfur/Li_2_S and low sulfur areal loading associated high electrolyte/sulfur ratio (E/S) are underlying reasons that account for these challenges [3]. Great efforts had been made in the past decades trying to develop advanced Li–S battery with stable performance and high energy density, including new sulfur host material exploration [4–6], electrolyte formula optimization [7, 8], and robust electrode/cell architecture construction [9–11]. Among them, functional sulfur host materials with variety microstructures and surface properties are the main research streams that were anticipated to entrap polysulfide in the cathode [12, 13]. Indeed, the polysulfide physi-/chemisorption strategies are promising to suppress the detrimental shuttle effect [14–16]. These approaches have shortened the gap between research and application for Li–S batteries.

We discovered that the MXene phase, a fascinating two-dimensional material with decent conductivity, demonstrating strong Lewis acid-based interaction with lithium polysulfides by metal–sulfur (S–Ti–C) binding at the interface [4]. We further elucidated that the cleavage of the hydroxyl terminal group from MXene contributes to retardation of polysulfide dissolution by another well-established mechanism—the thiosulfate-polythionate conversion [17, 18]. Benefitted from the high electric conductivity and rich surface properties that could synergistically improve the electron transport properties of the sulfur electrode and provide chemical interactions with polysulfides, MXene is regarded as one of the most promising sulfur host candidates [19]. Moreover, MXene has also been applied to various batteries due to the excellent electrochemical performance [20, 21].

Note that solely relying on these polysulfide confinement strategies in practical cells could partially alleviate the shuttling effect because of the relatively low fraction of the electrochemically inert host material in the electrode. Insertion of interlayers between the separator and the cathode has been demonstrated to further alleviate the polysulfide shuttle [22–24]; however, debates have been taken place because this interlayer inevitably sacrifices the volumetric/gravimetric density of the battery. MXene nanosheets have been successfully applied as the interlayer to improve the stability of the Li–S battery [25, 26]. The potential of the interlayer approach towards practical application relies on how thick and what a small weight ratio could the interlayer be manufactured.

While the techno-economic analysis suggests that cathodes of Li–S battery with areal capacity of 4 mAh cm^−2^ are necessary to compete with state-of-the-art Li-ion technology [12], the thick electrodes are challenged by volume expansion and poor kinetics. Designing architectures that could accommodate a large amount of sulfur and the corresponding volume change, effectively entrap the polysulfides, and maintain the ionic and electronic conducting pathways over cycling are highly desired [14, 27]. Optimized architectures such as microporous electrode [28, 29], three-dimensional framework electrodes [30, 31], and cross-linking sulfur hosts [32, 33] are pleasurable alternative approach towards high-performance Li–S batteries.

Herein, we performed comprehensive material design and cell architecture construction based on the above MXene phase (Ti_3_C_2_T_*x*_ nanosheets), aiming at realizing stable cycling performance of Li–S battery with high sulfur areal loading. The intrinsic negatively charged MXene nanosheets were assembled to the positively charged Ketjen black/sulfur (KB/S) particles to construct the interwoven KB/S@Ti_3_C_2_T_*x*_ composite. While the Ketjen black core improves the electric conductivity for the sulfur, the Ti_3_C_2_T_*x*_ guarantees physi-/chemisorption of the soluble polysulfide species and further enhances the in-plane conductivity. More importantly, the self-assembled secondary particle structure benefits the structure integrality bearing the volume expansion/shrinkage of the sulfur electrode. The KB@Ti_3_C_2_T_*x*_ prepared by similar self-assembly approach was coated on commercial separator to further retard the polysulfide that possibly escaped from the cathode. The KB@Ti_3_C_2_T_*x*_ interlayer, only 0.28 mg cm^−2^ in areal loading and 3 μm in thickness, accounts a little contribution to the volume and the mass of the thick electrode; thus, the effect on the energy density is minimal. By coupling the robust KB@Ti_3_C_2_T_*x*_ cathode and the effective KB@Ti_3_C_2_T_*x*_ modified separator, we achieved a stable Li–S battery with high sulfur areal loading (5.6 mg cm^−2^) and high areal capacity (6.4 mAh cm^−2^) at relatively lean electrolyte.

## Experimental Section

### Synthesis of Ti_3_C_2_T_*x*_

Ti_3_C_2_T_*x*_ colloidal solution was synthesized by liquid-phase delamination of Ti_3_AlC_2_ powder [34]. Typically, 2 g LiF was added to 40 mL of 9 M HCl and stirred for 10 min, followed by slowly addition of 2 g Ti_3_AlC_2_. The etching reaction was held at room temperature for 24 h under magnetic stirring. The resultant was washed and centrifuged with deionized water until the pH of the supernatant was approximately 6. Subsequently, the precipitate was mixed with deionized water and sonicated under Ar-protected environment for 1 h and centrifuged at 3500 rpm for 1 h to remove any large particles.

### Synthesis of KB/S Composite

The KB/S composite was produced by liquid phase method as reported elsewhere [35]. Briefly, 1.8 g sulfur and 0.25 g Ketjen black (KB) were added into 50 mL ethanediamine (EDA) with stirring for 20 min, then 8% HNO_3_ solution was slowly added into the mixture until the pH ≈ 6. After magnetic stirring for 2 h, the KB/S composite was collected by filtering and washing with DI water followed by drying at 60 °C for 24 h.

### Synthesis of KB/S@Ti_3_C_2_T_*x*_ Composite

0.155 g of KB/S composite and 0.3 g poly(ethylenimine) were dispersed evenly in 150 mL DI water by sonication. Next, the solid was collected by vacuum filtration and then re-dispersed in 150 mL water. Ti_3_C_2_T_*x*_ with the concentration of 0.5 mg L^−1^ was added to the above suspension and stirred for 2 h. The product was washed with DI water and dried in vacuum for 24 h.

### Preparation of KB@Ti_3_C_2_T_*x*_-coated Separators

The synthesis method of the KB@Ti_3_C_2_T_*x*_ composite (KB:Ti_3_C_2_T_*x*_ = 2:8, w/w) is similar to the KB/S@Ti_3_C_2_T_*x*_ composite. The KB@Ti_3_C_2_T_*x*_ was added to 0.2 wt% polyvinylidene fluoride (PVDF) solution with N-methyl-2-pyrrolidinone solution (NMP) as solvent where a ratio of KB@Ti_3_C_2_T_*x*_/PVDF was 9:1. The slurry was then cast onto commercial separator (Celgard 2500) and dried in a vacuum oven at 50 °C overnight.

### Material Characterization

XRD measurements were carried out on X-ray diffraction (XRD, Rigaku RINT-2000) with Cu Kα radiation. SEM studies were carried out on a JSM-6700Femission SEM instrument. TEM studies were carried out on high-resolution transmission electron microscopy (HRTEM Philips TecnaiF20, 200 kV). Thermogravimetric analysis conducted on DTG-60 instrument was used to determine the sulfur content of the composite under an argon flow. Brunauer–Emmett–Teller (BET) measurements were carried out on a JW-BK200C surface area analyzer operating at nitrogen atmosphere and the adsorption temperature of 77 k. Samples were dried for 24 h in vacuum at 60 °C prior to the characterization.

### Electrochemical Measurements

The electrodes were fabricated by casting a slurry containing 80 wt% active material (i.e., KB/S or KB/S@Ti_3_C_2_T_*x*_), 5 wt% super P, 5 wt% CNT, and 10 wt% La133 on carbon paper (HCP020N, Shanghai Hesen) and dried at 60 °C. The diameter of the electrode is 10 mm in diameter, and the sulfur loading is about 1.5 mg cm^−2^. CR2032-type coin cells were assembled in an argon-filled glove box. Lithium foil was used as the anode, and 1 M LiTFSI in 1, 2-dimethoxyethane (DME): 1, 3-dioxolane (DOL) (1:1 vol) with 2 wt% LiNO_3_ additive was used as the electrolyte. The batteries were galvanostatic charge/discharge cycled between 1.7 and 2.8 V on LAND battery tester. Cyclic voltammetry (CV) tests were performed on a VMP-3 workstation (Bio-logic) at a scan rate of 0.1 mV s^−1^ between 1.5 and 3.0 V. Electrochemical impedance spectroscopy (EIS) tests were carried out at a frequency range of 10 mHz to 100 kHz on the same workstation.

### Polysulfides Adsorption Test

Polysulfides solution (Li_2_S_6_) was prepared by mixing lithium sulfides (Li_2_S) and sulfur powder at a molar ratio of 5:1 in DME followed by magnetic stirring at 60 °C. Then 15 mg of the samples (KB or KB@Ti_3_C_2_T_*x*_) were added to 5 mL of 1.5 mM Li_2_S_6_ in DME.

## Results and Discussion

### Robust KB/S@Ti_3_C_2_T_*x*_ Composite Bears Volume Change

**Fig. 1:**
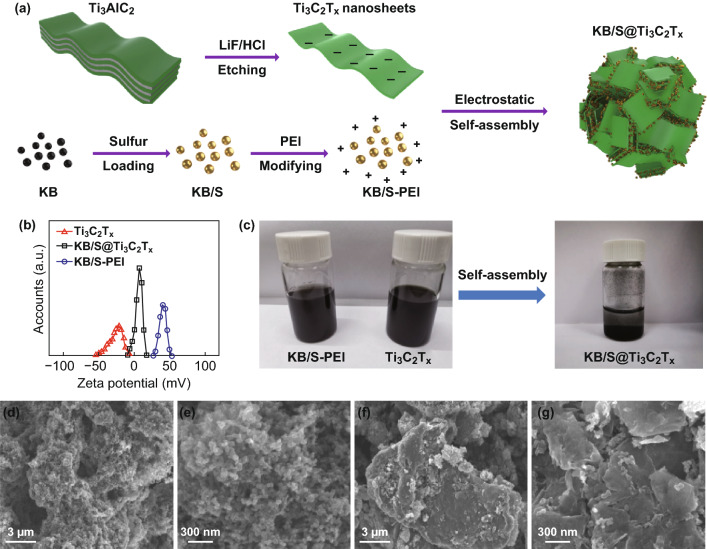
**a** Schematic illustration of the fabrication of the KB/S@Ti_3_C_2_T_*x*_ composite. **b** Zeta potential of Ti_3_C_2_T_*x*_ nanosheets, KB/S-PEI and KB/S@Ti_3_C_2_T_*x*_. **c** Digital photographs of KB/S-PEI, Ti_3_C_2_T_*x*_, and KB/S@Ti_3_C_2_T_*x*_ aqueous suspension. SEM images of KB/S (**d**, **e**) and KB/S@Ti_3_C_2_T_*x*_ (**f**, **g**)

MXene is a negatively charged material due to its surface functional groups [36], which is widely used to synthesize the nanosized composites by self-assembly through electrostatic interaction with the positively charged nanoscale components [37]. We used this self-assembly approach to construct an interwoven structure of the KB/S@Ti_3_C_2_T_*x*_ composites, as schematically illustrated in Fig. 1a.

The KB/S core was firstly prepared by a liquid phase method instead of melt-diffusion to avoid uneven sulfur distribution at high sulfur content (see Methods). The KB/S particles were then positively charged by poly(ethyleneimine) (PEI) in aqueous solution, due to the positive charge from the high density of amines [38]. As shown in Fig. 1b, the zeta potential of the Ti_3_C_2_T_*x*_ nanosheets and the PEI-decorated KB/S was measured to be − 21 and 40 mV, respectively. Before mixing, the individual KB/S and Ti_3_C_2_T_*x*_ aqueous solution are homogenous suspensions due to charge repulsion; however, they precipitated rapidly upon mixing through electrostatic interaction with the zeta potential neutralized to 7 mV (Fig. 1c). SEM images (Fig. 1d, e) reveal the homogenous KB/S composites with interconnected nano-size KB/S particles in the absence of aggregation. The sulfur content of the KB/S composite is determined to be 82 wt% (Fig. S1). KB/S composite was uniformly assembled to the MXene nanosheets due to electrostatic interaction and further aggregated to form secondary particles with a size of about 10 microns (Fig. 1f, g). TGA reveals the sulfur content in the final composite is about 60 wt% (Fig. S1). EDS elemental mapping (Fig. S2) indicates the homogeneous distribution of sulfur, carbon and Ti_3_C_2_T_*x*_ nanosheets in the composite. The KB/S@Ti_3_C_2_T_*x*_ composite has the advantage of highly conductive core that is provided by the KB carbon with unique branched structure and high surface area [39], while the MXene guarantees physi-/chemisorption for the soluble polysulfide species and further enhances the in-plane conductivity [40]. Most importantly, this interwoven structure benefits the structure integrality for the volume expansion/shrinkage of the sulfur electrode, as will be discussed next. The KB/S@Ti_3_C_2_T_*x*_ is compromised by 60 wt% sulfur, 13 wt% KB and 27 wt% Ti_3_C_2_T_*x*_ nanosheets. The TEM image and X-ray diffraction pattern of the Ti_3_C_2_T_*x*_ are shown in Fig. S3. The TEM image shows the as-prepared Ti_3_C_2_T_*x*_ nanosheets are almost monolayer in thickness with several micron in 2-D size. XRD pattern proves the Ti_3_C_2_T_*x*_ are well exfoliated [9, 25]. Figure S4 shows the XRD patterns of KB/S and KB/S@Ti_3_C_2_T_*x*_, proving the sulfur maintains in the orthorhombic phase, and the MXene (002) plane is clearly shown in the KB/S@Ti_3_C_2_T_*x*_ composite.

It is well accepted that the full conversion of sulfur to Li_2_S results in a volumetric expansion over 80%, which brought huge challenges to the long-term cycling of Li–S battery [41]. It attributes to the mechanical degradation of the conductive network in electrode, leading to irreversible structural destruction with a decrease in mechanical integrity and the subsequent rapid capacity decay, especially in the case of high-sulfur-loaded electrodes [42]. Materials engineering to build smart cathode architectures that allow high sulfur loading and accommodate the corresponding volume change, while maintain the structural integrity and the ionic and electric conducting pathways, is highly desired [13].

**Fig. 2:**
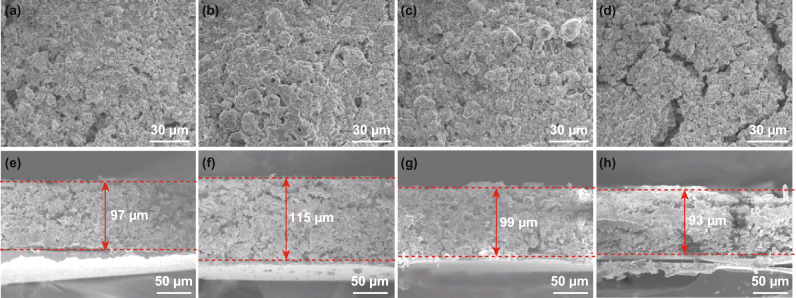
Comparison of the sulfur electrode by SEM. Surface images of **a** fresh KB/S@Ti_3_C_2_T_*x*_ electrode, **b** KB/S@Ti_3_C_2_T_*x*_ electrode after 10 cycles, **c** fresh KB/S electrode, **d** KB/S electrode after 10 cycles. Cross-sectional images **e** before cycle and **f** after 10 cycles of the KB/S@Ti_3_C_2_T_*x*_ electrode, **g** fresh KB/S electrode and **h** after 10 cycles of the KB/S electrode

We systematically evaluated the electrode structure evolution during cycling. Figure 2 compares the SEM images of the KB/S and the KB/S@Ti_3_C_2_T_*x*_ electrode, in the initiate state and in the cycled state, respectively. Both KB/S and the KB/S@Ti_3_C_2_T_*x*_ particles are interconnected compactly in their fresh electrodes, as clearly demonstrated by the surface and the cross-sectional SEM images (Fig. 2a, c, e, g). After 10 cycles, conspicuous damage and cracks occurred on the surface of KB/S cathode (Fig. 2d). In contrast, no significant change is detected for the KB/S@Ti_3_C_2_T_*x*_ electrode (Fig. 2b). The cross-sectional SEM images give more detailed information about the electrode architecture. Both electrodes have identical thickness before cycling. The KB/S@Ti_3_C_2_T_*x*_ electrode slightly expands about 18% with tightly connected particles (Fig. 2e, f), while the KB/S electrode shrinks upon charge and leaves cracks and holes in the electrode (Fig. 2g, h). We assume polysulfide dissolution accounts for the shrinkage of the KB/S electrode, resulting in active material loss and leaves holes in the electrode. The difference reflects that the KB/S@Ti_3_C_2_T_*x*_ composite has the capacity to maintain the active materials in the electrode owing to both physical and chemical confinement provided by the MXene nanosheets. Meanwhile, the interwoven structure could accommodate the volume change, thus effectively maintaining the electrode integrity during cycling.

**Fig. 3:**
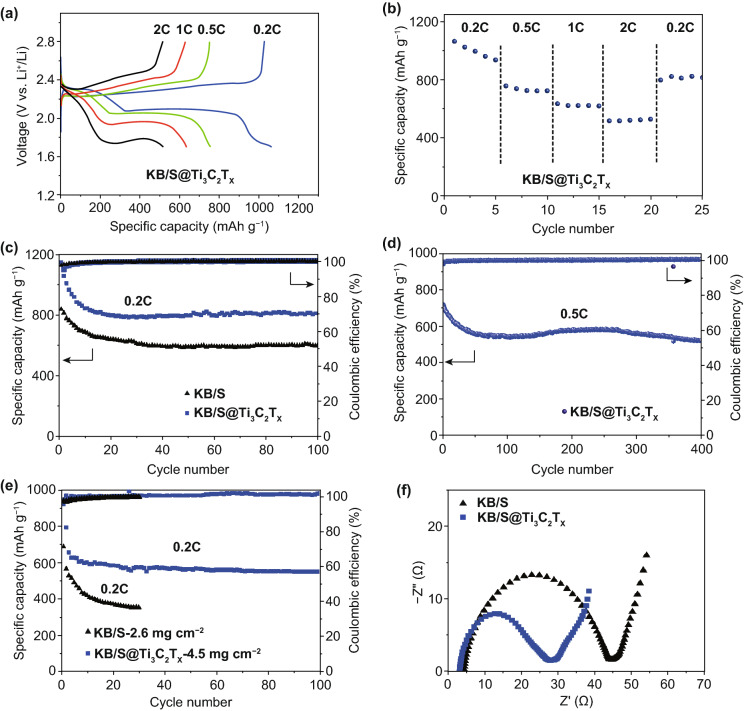
**a** Voltage profiles of the KB/S@Ti_3_C_2_T_*x*_ at various rates ranging from 0.2 to 2 C. **b** Rate performances of KB/S@Ti_3_C_2_T_*x*_. **c** Cycling performance of KB/S and KB/S@Ti_3_C_2_T_*x*_ at 0.2C. **d** Long-term cycling of KB/S@Ti_3_C_2_T_*x*_ at 0.5 C. **e** Cycling performance of KB/S and KB/S@Ti_3_C_2_T_*x*_ with high sulfur loading of 2.6 mg cm^−2^ and 4.5 mg cm^−2^ at 0.2 C, respectively. **f** Nyquist plots of the KB/S and KB/S@Ti_3_C_2_T_*x*_ electrodes

The electrochemical performance was evaluated in coin cells with conventional electrolyte (1 M LiTFSI in DOL-DME with 2 wt% LiNO_3_). Figure 3a shows the charge/discharge curves of KB/S@Ti_3_C_2_T_*x*_ cathode at different current densities between 1.7 and 2.8 V. Two distinct voltage plateaus are clearly presented with the higher one corresponding to high-order polysulfide formation and the lower one referring to solid Li_2_S nucleation, respectively. The polarization between charge and discharge process is increasing as the function of the increased current density. Accordingly, the discharge plateaus are shifted to lower potential. The KB/S cathode shows similar charge/discharge curves; however, the polarization is much greater upon increasing the current density. For example, the KB/S@Ti_3_C_2_T_*x*_ electrode has two distinct plateau, but the KB/S electrode only exhibits one slopping curve at 2 C (Figs. 3a, and S5). The difference in polarization indicates the sulfur redox kinetic was improved in KB/S@Ti_3_C_2_T_*x*_ electrode, which was further supported by the lower charge-transfer resistance in the electrochemical impedance spectrum (EIS) (Fig. 3f). Obviously, the MXene nanosheets assembled to KB/S particles provide better electric conductivity of the composite, facilitating the charge transfer process and promote the redox kinetics.

We witness a superior rate performance of the KB/S@Ti_3_C_2_T_*x*_ electrode (Fig. 3b) than that of the KB/S electrode (Fig. S6). The KB/S@Ti_3_C_2_T_*x*_ electrode delivers high specific capacities of 1062, 756, 634, and 517 mAh g^−1^ at the rate of 0.2, 0.5, 1, and 2 C, respectively. However, the KB/S cathode showed lower discharge capacity of 622, 480, 358, 156 mAh g^−1^ at the same rates, respectively (Fig. S6). The KB/S@Ti_3_C_2_T_*x*_ cathode conveys better cycling performance than the KB/S electrode, as exemplified by long-term cycling at 0.2 C (Fig. 3c). After 100 cycles, the discharge capacity of the KB/S@Ti_3_C_2_T_*x*_ electrode was 812 mAh g^−1^, while that of the KB/S electrode was only 603 mAh g^−1^ in the same test condition (Fig. 3c). Longer cycling was conducted at 0.5 C, showing a very stable performance over 400 cycles (Fig. 3d). We attribute the improved electrochemical performance of the KB/S@Ti_3_C_2_T_*x*_ electrode to the optimized interwoven structure, which provides better conductivity while suppresses the polysulfide dissolution via physical and chemical confinement. Given the fact that the KB/S@Ti_3_C_2_T_*x*_ electrode provides enhanced robustness of the structure integrity as discussed above, we are allowed to cycle the cell with higher sulfur areal loading. Figure 3e shows the KB/S@Ti_3_C_2_T_*x*_ electrode with sulfur loading of 4.5 mg cm^−2^. The battery was activated at 0.05 C for the first two cycles followed by long-term cycling at 0.2 C. The discharge capacities are 920 and 655 mAh g^−1^ at 0.05 and 0.2 C, respectively. The capacity retains 551 mAh g^−1^ after 100 cycles. In sharp contrast, KB/S batteries exhibited lower capacity and decayed rapidly even at lower sulfur areal loading (2.6 mg cm^−2^), as shown in Fig. 3e.

### Effective KB@Ti_3_C_2_T_*x*_ Further Improves the Sulfur Utilization

Though effective, the specific capacity that KB@Ti_3_C_2_T_*x*_ composite delivered is not satisfactory, as discussed above. Further improvement of the sulfur utilization in the composite depends on the extent of the polysulfide shuttle that could be suppressed [43]. The combination of physical and chemical confinement of the polysulfide in the KB@Ti_3_C_2_T_*x*_ composite, however, is not sufficient due to two reasons. First, the electrostatic interaction that accounts for the self-assembly of the KB/S@Ti_3_C_2_T_*x*_ composite is not as strong as the covalent bonds, which leaves space at the junction of the two overlapped MXene nanosheets for electrolyte penetration but also allows polysulfide escape. Second, the chemical adsorption of polysulfide is normally based on ‘monolayer’ interaction with the interface, which is not sufficient to trap the polysulfide, especially at a high sulfur content. Though the polysulfide adsorption capacity by MXene is evaluated to be higher than most of the sulfur host materials [14], such a low MXene-to-sulfur ratio (3:7 by weight) indicates possible polysulfide escape. Insertion of interlayers between the separator and the cathode has been demonstrated to be a promising approach to further alleviate polysulfide shuttle [44]. MXene nanosheets have been successfully applied as the interlayer to improve the stability of the Li–S battery [45]. In this section, we designed the KB@Ti_3_C_2_T_*x*_ coated commercial polypropylene (PP) separators and try to fabricate a lithium sulfur battery with high sulfur areal loading, in the combination of the above-studied KB/S@Ti_3_C_2_T_*x*_ electrodes.

**Fig. 4:**
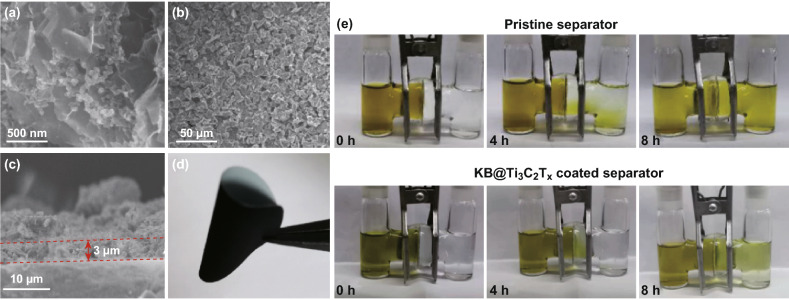
**a** SEM image of KB@Ti_3_C_2_T_*x*_. **b** SEM image of KB@Ti_3_C_2_T_*x*_-coated separator. **c** Cross-sectional SEM image of KB@Ti_3_C_2_T_*x*_-coated separator. **d** Photograph of the KB@Ti_3_C_2_T_*x*_-coated separator. **e** Polysulfides permeation measurements with the pristine PP separator and KB@Ti_3_C_2_T_*x*_-coated separator

The merit of the KB carbon in the interlayer is to prevent the restacking of the MXene nanosheets so as to expose most of the active sites on MXene. The composite (KB:Ti_3_C_2_T_*x*_ = 2:8, w/w) was prepared by similar self-assembly method discussed above without sulfur in the precursor. The zeta potentials of the composite suggest the successful self-assembly between KB and Ti_3_C_2_ nanosheets (Fig. S7). The SEM image shows the KB@Ti_3_C_2_T_*x*_ composite has an interwoven MXene matrix with homogenously dispersed KB carbon (Fig. 4a). Due to higher Ti_3_C_2_T_*x*_ ratio, the KB@Ti_3_C_2_T_*x*_ composite is not in spherical morphology as the KB/S@Ti_3_C_2_T_*x*_ composite. The specific surface area of the KB@MXene composite is 131 m^2^ g^−1^, as measured by Nitrogen adsorption isotherms (Fig. S8). The increase in surface area of the KB@MXene composite indicates the MXene nanosheets are greatly prevented from re-stacking to promise abundant exposed active sites. Polysulfide adsorption measurements show the strong affinity of KB@Ti_3_C_2_T_*x*_ toward polysulfides, while KB carbon only has minor polysulfide adsorption (Fig. S9). The composite was casted onto separators by doctor blade. SEM image shows the KB@Ti_3_C_2_T_*x*_ micron particles are uniformly coated on the surface of separator (Fig. 4b). The mass loading of KB@T_i3_C_2_T_*x*_ is about 0.28 mg cm^−2^. The thickness is about 3 μm, accounting for only 3% of the thickness of the cathode (Fig. 4c). The KB@Ti_3_C_2_T_*x*_ interlayer, much thinner than most of the reported interlayers [6, 46, 47], is too thin to significantly affect the volumetric/gravimetric energy density of the Li–S batteries [48]. A comparison with the literatures about the thickness and the areal mass of the interlayer in Li–S batteries is summarized in Table S1. The KB@Ti_3_C_2_T_*x*_ interlayer shows excellent electrochemical performance even at low areal mass and thin thickness. The as-prepared KB@Ti_3_C_2_T_*x*_-coated separator maintains the flexibility and excellent mechanical strength, as demonstrated by the foldable shape (Fig. 4d).

In order to verify the KB@Ti_3_C_2_T_*x*_-coated separator has the capacity to restrain the polysulfide shuttle, visualized H-type glass cells with 1 mM Li_2_S_6_ solution were assembled (Fig. 4e). Polysulfides diffused across the unmodified PP separator quickly, accompanied by color changed of the counter container of the H-cells from colorless to pale yellow within 4 h, indicating uncontrolled polysulfide diffusion. In sharp contrast, the cell with KB@Ti_3_C_2_T_*x*_-coated separator does not have visual difference in 4 h; the solution in the right-side container only started to turn light pale yellow after 8 h. We expect significant improvements to the Li–S battery when using this polysulfide diffusion retardant KB@Ti_3_C_2_T_*x*_-coated separator. In addition, we studied the Li metal morphology after cycled in Li–S batteries. The SEM image shows a rouge surface of the lithium anode with severe corrosion in the cell with regular separator, whereas the Li anode KB@Ti_3_C_2_T_*x*_-coated separator is relatively uniform and smooth surface (Fig. S10). The differences could be ascribed to corrosion of the Li metal by polysulfides.

**Fig. 5:**
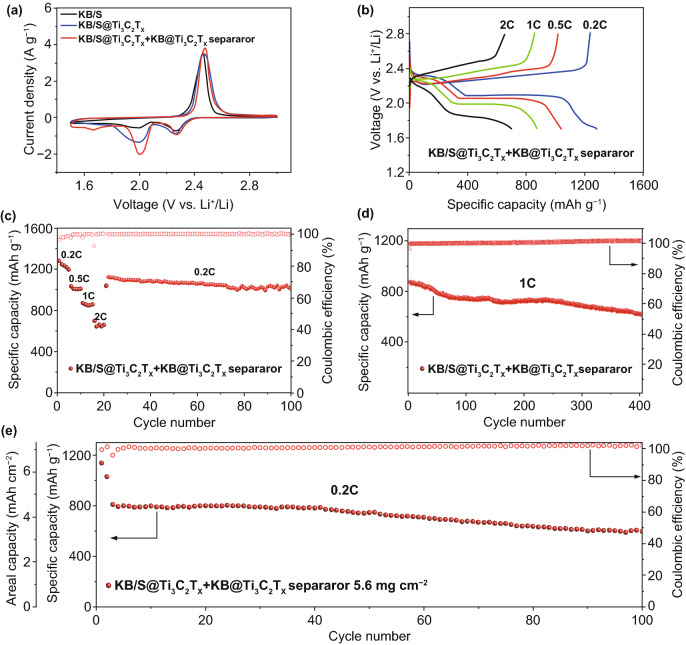
**a** CV profiles of KB/S and KB/S@Ti_3_C_2_T_*x*_, with the pristine separators, and the KB/S@Ti_3_C_2_T_*x*_ electrode with the KB@Ti_3_C_2_T_*x*_ modified separator. **b** Voltage profiles of the KB/S@Ti_3_C_2_T_*x*_ cell with the KB@Ti_3_C_2_T_*x*_ modified separator at various rates ranging from 0.2 to 2 C. **c** Rate performances, **d** long-term cycling performance at 1 C of the KB/S@Ti_3_C_2_T_*x*_ electrode with the KB@Ti_3_C_2_T_*x*_ separator. **e** Cycling performance with high sulfur loading of 5.6 mg cm^−2^ at 0.2 C

Figure 5 shows the impacts of the KB@Ti_3_C_2_T_*x*_ modified separator to the electrochemical performance of the Li–S batteries. Comparisons are made between the cells based on the KB/S@Ti_3_C_2_T_*x*_ electrode with and without the modified separators. The cyclic voltammetry (CV) measurements convey that the modified separator promoted the redox kinetics of the sulfur electrode (Fig. 5a). The redox potentials of the three cells are identical: two cathodic peaks at about 2.3 and 2.0 V which are corresponding to the formation of the soluble polysulfides (Li_2_S_4–8_) and insoluble polysulfides (Li_2_S_2_/Li_2_S), respectively. The anodic peak at about 2.5 V is attributed to the oxidation process of polysulfides to sulfur. It is apparently that the cathodic peak current is increased by the KB@Ti_3_C_2_T_*x*_-coated separator. We believe that MXene in the interlayer restrains the escape of polysulfide during the charge/discharge process and offers extra reaction sites for active materials thus additional electron flow is gained. The modified separator also decreases the resistance of the cell as shown by the Nyquist plots (Fig. S11). The quasi-semicircle at the high-frequency region is attributed to the charge transfer impedance (*R*_*ct*_) [49]. This indicates that the KB@Ti_3_C_2_T_*x*_ interlayer can remarkably activate the electrochemical reaction and promote the charge transfer property. The EIS of different Li–S cells are measured after cycling, as shown in Fig. S12. We further fitted the curves to compare the change of *R*_*sei*_ and *R*_*ct*_ during cycling by using the equivalent circuit shown in Fig. S12 d, e. The fitted values are listed in Table S2. Apparently, the *R*_*ct*_ of the KB/S@Ti_3_C_2_T_*x*_ electrode and KB/S@Ti_3_C_2_T_*x*_ electrode with KB@Ti_3_C_2_T_*x*_ interlayer are smaller than the KB/S electrode, which is owing to the high conductivity of MXene. The *R*_*sei*_ and *R*_*ct*_ of KB/S electrode during cycling, indicative of serious polysulfide dissolution which forms passivation layer on the electrode surface. However, *R*_*sei*_ of the KB/S@Ti_3_C_2_T_*x*_ electrode or the KB/S@Ti_3_C_2_T_*x*_ electrode with KB@Ti_3_C_2_T_*x*_ interlayer is decreasing during cycling, suggesting the escape of polysulfide is impeded by the secondary particle structure and the interlayer.

A straight conclusion could be made that the KB@Ti_3_C_2_T_*x*_ modified separator improves the specific capacity of the battery, i.e., 1281 versus 1062 mAh g^−1^ with the pristine separator at 0.2 C. (Figs. 3a, 5b). This is consistent with the CV curves in Fig. 5a, in which the modified separator activates the dissolved polysulfide in the electrolyte; thus, a higher specific capacity was delivered. The cells with the KB@Ti_3_C_2_T_*x*_ modified separator also demonstrate better rate performance, as shown in Fig. 5c. Remarkably, it delivers 1014 mAh g^−1^ after 80 cycles when the current density is adjusted back to 0.2 C. The cells with the KB@Ti_3_C_2_T_*x*_ modified separator also have better cycling performance at 1 C, as shown in Fig. 5d. The cell displays an initial discharge capacity of 880 mAh g^−1^, and still retains a considerable capacity of 629 mAh g^−1^ after 400 cycles, corresponding to a decay of 0.071% per cycle. In order to illustrate the role of KB@Ti_3_C_2_T_*X*_ interlayer, we paired the KB/S electrode with the KB@Ti_3_C_2_T_*X*_ interlayer, as shown in Fig. S13. The cell displays an initial discharge capacity of 754 mAh g^−1^ at 1 C, and still retains a considerable capacity of 514 mAh g^−1^ after 400 cycles. The improvement by using the KB@Ti_3_C_2_T_*X*_ interlayer compared to the cell with regular separators (Fig. 3c), further indicating that KB@Ti_3_C_2_T_*X*_ interlayer could hinder the polysulfide shuttle and improves sulfur utilization. We compared the performance with other publications with low sulfur loading in Table S3, because most of the available publications are reported with sulfur loading < 2 mg cm^−2^. It shows that the comprehensive engineering of the MXene nanosheets afford to a high capacity and high capacity retention for long-term cycling.

Benefiting from the promising microstructural KB/S@Ti_3_C_2_T_*x*_ electrode and the effective interlayer, electrodes with higher sulfur loading were also tested (Fig. 5e). The sulfur areal loading is increased to 5.6 mg cm^−2^. Because of the minimal thickness of the interlayer, it does not absorb much electrolyte as other reported interlayer did. Thus it is possible to cycle the cell with relatively lean electrolyte of E/S ratio of 7 μL/mg. The batteries were activated by cycling at 0.05 C for the first two cycles, followed by 0.2 C in the subsequent cycles. The areal capacity is a critical parameter but always being neglected among the Li–S community [50]. Correspondingly, we have plotted area capacity and specific capacity in Fig. 5e. The discharge capacities of cell were 1137 mA h g^−1^ at 0.05 C and 810 mAh g^−1^ at 0.2 C, and the corresponding areal capacities were 6.4 mAh cm^−2^ and 4.5 mAh cm^−2^, respectively. The cell delivers about 600 mAh g^−1^ capacity after 100 cycles. We compared our work with other recent publications with high sulphur loading that involve MXenes in the electrodes or in the separators, as shown in Table S4. It shows that our work is superior or at least comparable to these references. This is owing to the interwoven KB@Ti_3_C_2_T_*X*_ composite as well as the modified separator, which not only provides superior polysulfide interaction and maintains the electrode integrality bearing the volume expansion/shrinkage, but also further retards the polysulfide cross-diffusion that possibly escaped from the cathode. We also believe this performance is competitive to the recently reported interlayer works with high sulfur areal loading. Moreover, our results are obtained at a more lean electrolyte condition [51].

## Conclusions

We have demonstrated a comprehensive design of material and cell construction aimed at achieving improved Li–S battery with high sulfur areal loading. The key materials are based on the fascinating MXene phase with known affinity to the polysulfide species. Material engineering for the sulfur host and the interlayer coated on separator were conducted through electrostatic self-assembly approach. The prepared KB/S@Ti_3_C_2_T_*x*_ has an interwoven structure in which the KB carbon core improves the electric conductivity, while the MXene nanosheets guarantees physi-/chemisorption of the soluble polysulfide species. More importantly, the interwoven structure benefits the structure integrality for the volume expansion/shrinkage of the sulfur electrode. To further retard the polysulfide that possibly escaped from the cathode, the KB@Ti_3_C_2_T_*x*_ was coated on the separator to function as an interlayer (about 3 μm). This interlayer does not sacrifice the volumetric/gravimetric energy density because of the minimal thickness and weight ratio; however, it retards and activates the dissolved polysulfide, improving the overall sulfur utilization. By coupling the robust KB/S@Ti_3_C_2_T_*x*_ cathode and the effective KB@Ti_3_C_2_T_*x*_ modified separator, we achieved a stable Li–S battery with high sulfur areal loading at relatively lean electrolyte. Further development of the high energy density Li–S batteries that cycled at *E*/*S* < 5 μL mg^−1^ via electrolyte formula and electrode architecture optimization is under investigation in our laboratory.

## Electronic supplementary material

Below is the link to the electronic supplementary material.

Supplementary material 1 (PDF 705 kb)
