# Supplementary material for: Comprehensive Design of the High-Sulfur-Loading Li–S Battery Based on MXene Nanosheets
Source: Nanomicro Lett. 2020 May 20;12:112. doi: 10.1007/s40820-020-00449-7 (PMC7770945; doi:10.1007/s40820-020-00449-7)
Supplement: Supplementary file 1 — Supplementary material 1 (PDF 705 kb) [file 40820_2020_449_MOESM1_ESM.pdf]

Supporting Information for

Comprehensive Design of the High Sulfur-Loading Li-S Battery Based on MXene Nanosheets

Shouzheng Zhang[1, 3, ‡], Ning Zhong[2, ‡], Xing Zhou[2], Mingjie Zhang[2], Xiangping Huang[3], Xuelin Yang[1, *], Ruijin Meng[2], Xiao Liang[2, *]

[1]College of Materials and Chemical Engineering, China Three Gorges University, 8 Daxue Road, Yichang, Hubei 443002, People's Republic of China

[2]State Key Laboratory of Chem/Biosensing and Chemometrics, College of Chemistry and Chemical Engineering, Hunan University, Changsha, 410082, People's. Republic of China

[3]College of Science, China Three Gorges University, 8 Daxue Road, Yichang, Hubei 443002, People's. Republic of China

[‡]Shouzheng Zhang and Ning Zhong contributed equally to this work

*Corresponding authors. E-mail: xlyang@ctgu.edu.cn (Xuelin Yang); xliang@hnu.edu.cn (Xiao Liang)

Supplementary Figures and Tables

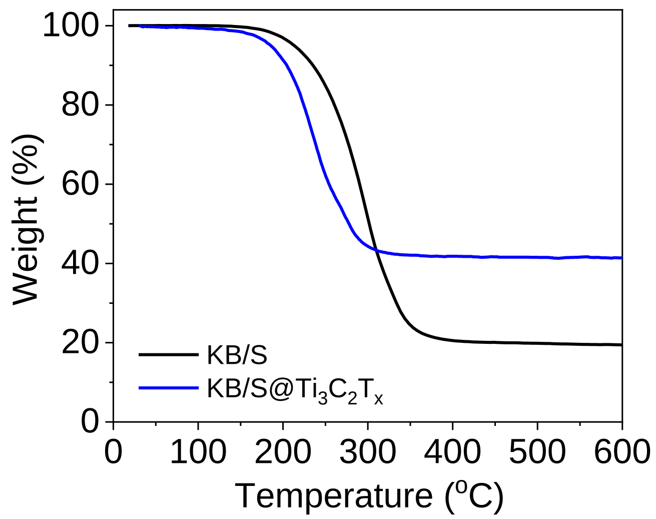

Fig. S1 TGA curves of the KB/S and KB/S@$Ti_3C_2T_x$

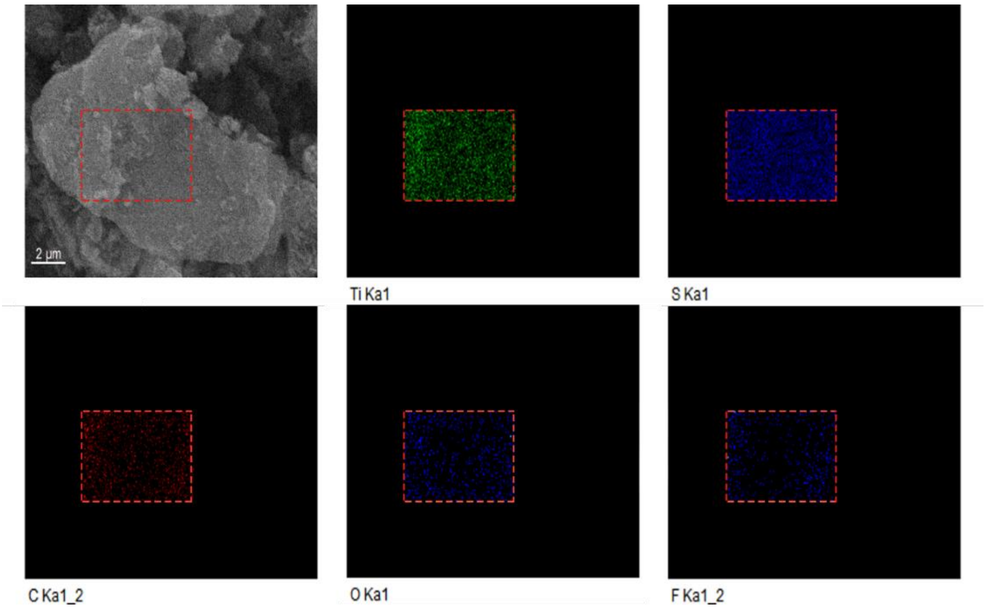

Fig. S2 SEM image and corresponding elemental mapping analysis of the KB/S@$Ti_3C_2T_x$

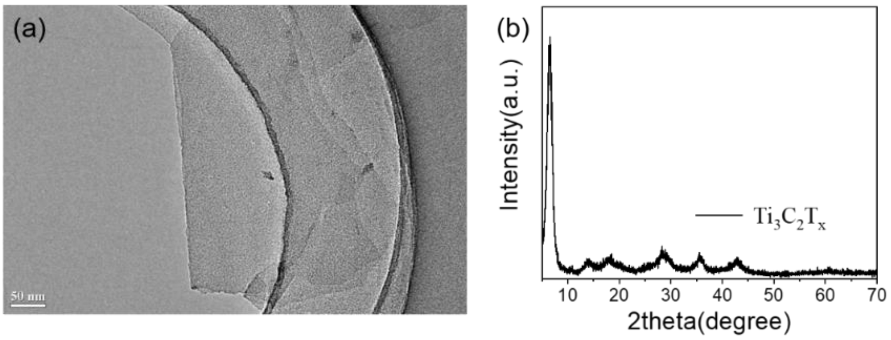

Fig. S3 The TEM image and X-ray diffraction pattern of the $Ti_3C_2T_x$

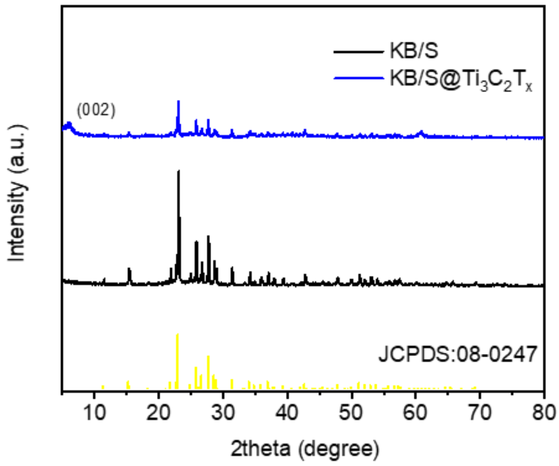

Fig. S4 XRD patterns of the KB/S composite and the KB/S@$Ti_3C_2T_x$

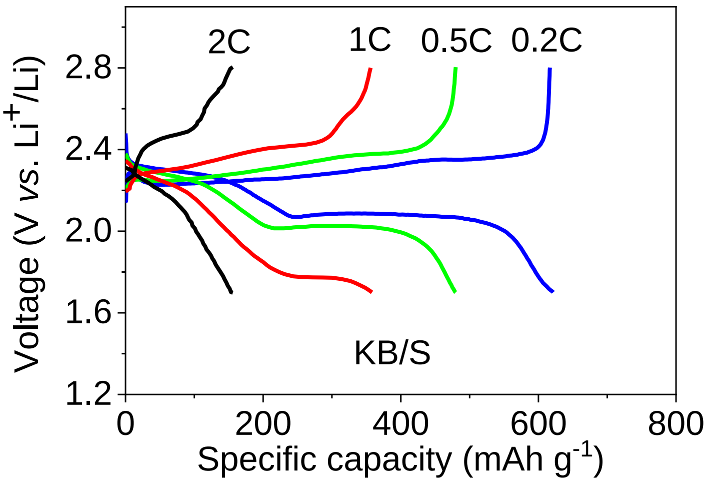

Fig. S5 Charge/discharge curves of of KB/S at various rates ranging from 0.2 to 2 C

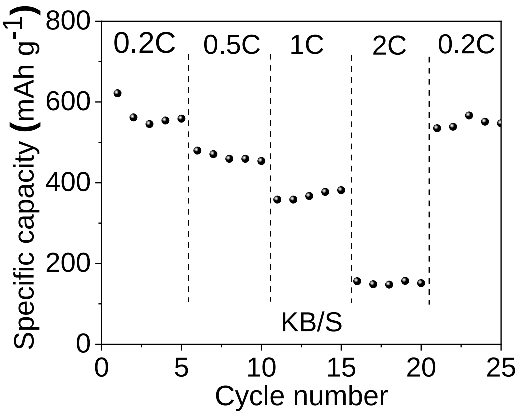

Fig. S6 Rate capability of KB/S electrode measured at different current densities from 0.2 to 2 C

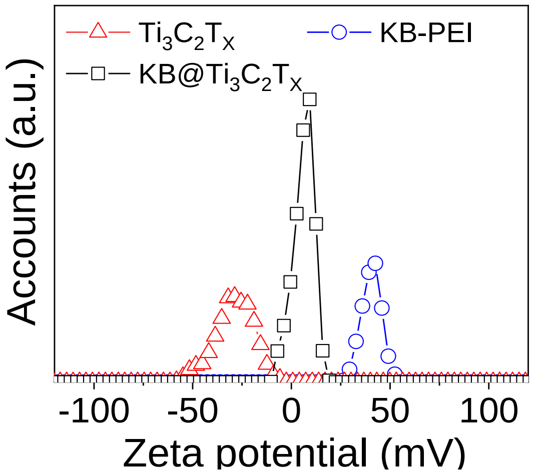

Fig. S7 Zeta potential of $Ti_3C_2T_x$ nanosheets, KB-PEI and KB@$Ti_3C_2T_x$

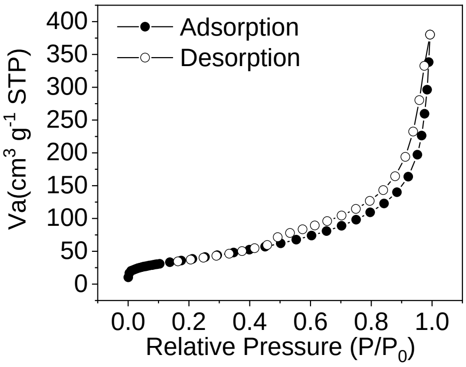

Fig. S8 N_2 adsorption-desorption isotherm of KB@$Ti_3C_2T_x$

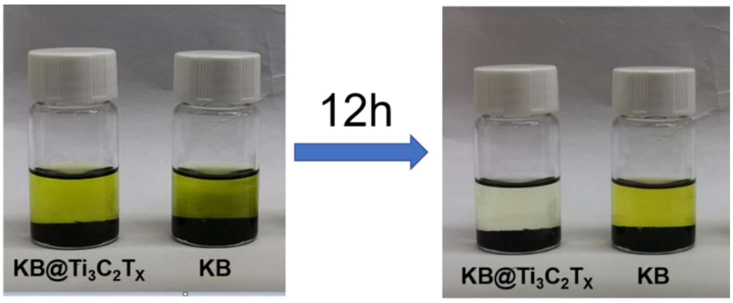

Fig. S9 Photograph of Li_2S_6 adsorption by KB@$Ti_3C_2T_x$ and KB in DME solution after 12 h

Table S1 A comparison with the literatures about the thickness and areal mass of the interlayer in Li-S batteries

Interlayer	Areal mass (mg cm^{-2})	Thickness (μm)	Performance	Refs.
PVDF-PEO/CB	0.6-0.7	10	751 mAh g^{-1} for 350 cycles, 0.3A g^{-1}.	[1]
NC@SA-Co and CNT-CNF hybrid network	0.45	10	787 mAh g^{-1} for 100 cycles, 0.5 C.	[2]
MoS_2/graphene	0.5	60	around 840 mAh g^{-1} in the initial 5 cycles, 1 A g^{-1}.<br>620 mAh g^{-1} for 200 cycles, 1 A g^{-1}.	[3]
RuO_2/mesoporous carbon	0.3	16	1276 mAh g^{-1} for the 1st cycle, 0.1 C.<br>665 mAh g^{-1} for the 300 cycles, 0.5 C.	[4]
CNT@ZIF	0.9	15	870 mAh g^{-1} for 100 cycles, 0.2 C.	[5]
KB@$Ti_3C_2T_x$	0.28	3	1281 mAh g^{-1} for the 1st cycle, 0.2 C.<br>1014 mAh g^{-1} for the 80 cycles, 0.2 C.<br>629 mAh g^{-1} for 400 cycles, 1 C.	This work

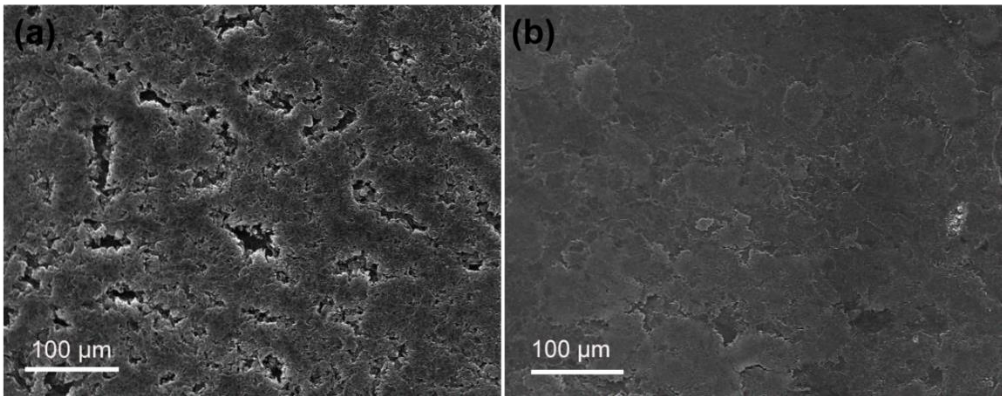

Fig. S10 The Li metal morphologies after cycling in different cells: (a) regular separator, (b) with KB@$Ti_3C_2T_x$ coated separator.

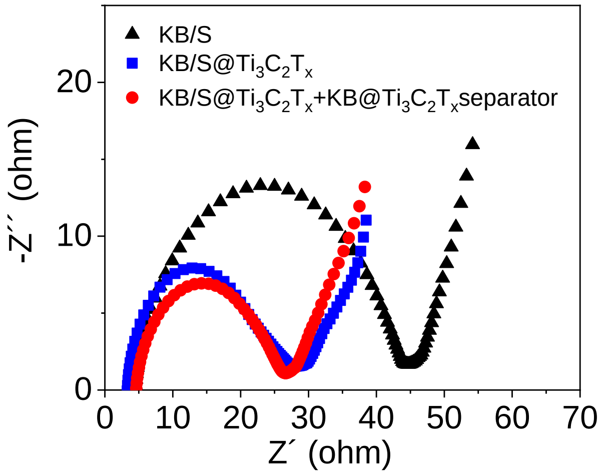

Fig. S11 Nyquist plots of KB/S, KB/S@$Ti_3C_2T_x$ electrodes with the pristine separators, and the KB/S@$Ti_3C_2T_x$ electrode with the KB@$Ti_3C_2T_x$ separator

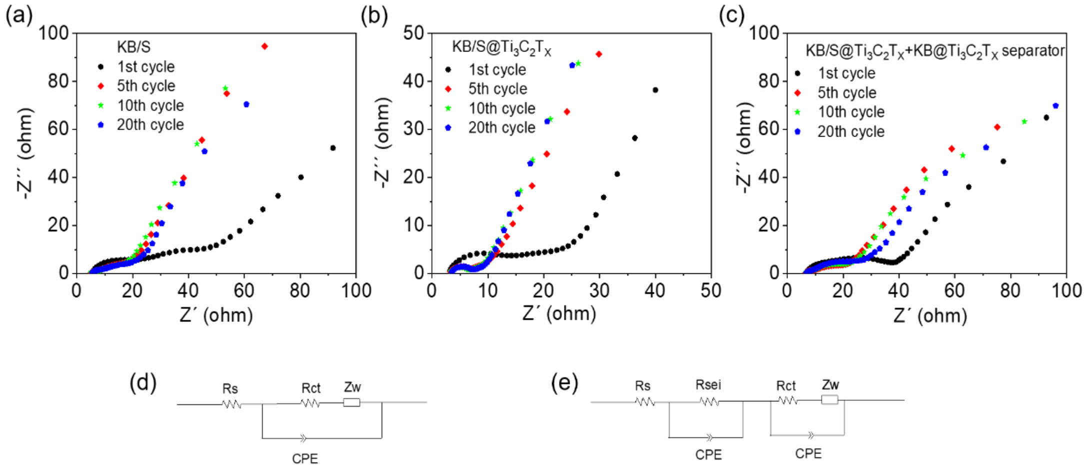

Fig. S12 EIS spectra of the Li−S cell (a) KB/S electrode, (b)KB/S@$Ti_3C_2T_x$ electrode and (c) KB/S@$Ti_3C_2T_x$ electrode with KB@$Ti_3C_2T_x$ coated separator after charging to 2.8 V, (d) and (e) Equivalent circuit for simulating the experimental data. The corresponding fitted resistances are listed in Table S2.

Table S2 Corresponding simulated resistances from the EIS fitting

Sample	Cycle	R_{sei} (Ω)	R_{ct} (Ω)
KB/S electrode	0	NO	38.3
	1st	97.7	54.6
	5th	215.7	42.1
	10th	370.1	26.7
	20th	660.9	36.4
KB/S@$Ti_3C_2T_X$ electrode	0	NO	22.78
	1st	23.1	3.2
	5th	16.0	3.8
	10th	14.4	0.9
	20th	13.67	1.9
KB/S@$Ti_3C_2T_x$ electrode with KB@$Ti_3C_2T_x$ coated	0	NO	21.62
	1st	36.1	2.8
	5th	16.7	0.9
	10th	17.5	1.0
	20th	15.5	1.1

Table S3 Comparison of cycling performance with different cathode materials and coating layers in Li-S batteries

Cathode materials	Materials on Separators	Sulfur loading (mg cm^{-2})	Electrochemical performance	Refs.
3D alkalized Ti_3C_2 MXene nanoribbon/S	Ti_3C_2	0.7-1	899 mAh g^{-1} for the 1st cycle, 0.5 C, 611 mAh g^{-1} for 50 cycles, 0.5 C.	[S6]
Carbon Black/S	Ti_3C_2	1.2	743.7 mAh g^{-1} for the 1st cycle, 1 C, 495 mAh g^{-1} for 500 cycles, 1 C.	[S7]
CNTs/S	$Ti_3C_2T_x$ /CNTs 10%	1.2	760 mAh g^{-1} for the 1st cycle, 1 C, 630 mAh g^{-1} for 200 cycles, 1 C.	[S8]
super P/S	Ti_3C_2 nanosheet /glass fiber	1.9	820 mAh g^{-1} for the 1st cycle, 0.5 A/g, 721 mAh g^{-1} for 100 cycles, 0.5 A/g.	[S9]
KB/S	$Ti_3C_2T_x$ eggshell	2.07	862 mAh g^{-1} for the 1st cycle, 1 C, 672 mAh g^{-1} for 150 cycles, 1 C.	[S10]
KB/S@$Ti_3C_2T_x$	KB@$Ti_3C_2T_x$	1.5	880 mAh g^{-1} for the 1st cycle,1 C, 629 mAh g^{-1} for 400 cycles, 1 C.	This work

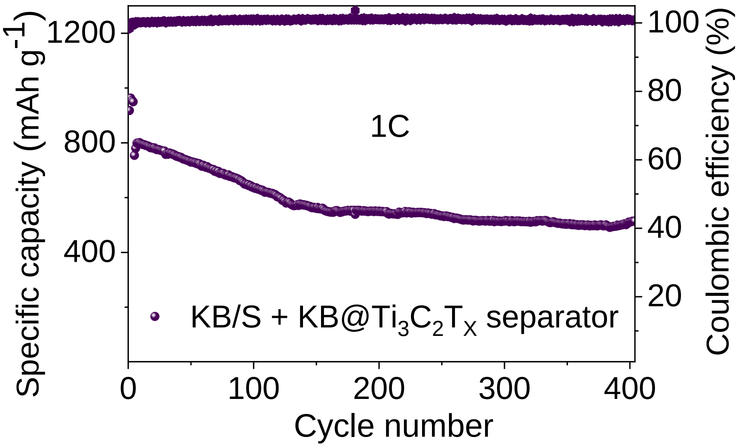

Fig. S13 Long-term cycling performance at 1 C of the KB/S electrode with the KB@$Ti_3C_2T_x$ separator

Table S4 Comparisons of the high-loading Li-S batteries with MXenes in the electrodes or in the separators

Cathode	Separator	Sulfur loading (mg cm^{-2})	Performance	Refs.
TiO_2 QDs@ MXene/S	blank	5.5	533 mAh g^{-1} for 100 cycles, 0.2 C.	[S11]
KB/S	$Ti_3C_2T_x$ eggshell	2.07	862 mAh g^{-1} for the 1st cycle, 1 C, 672 mAh g^{-1} for 150 cycles, 1 C.	[S12]
S/Carbon Black	Ti_3C_2	2.8	850.9 mAh g^{-1} for 30 cycles, 0.2 C.	[S13]
Ti_3C_2/C-S	blank	2.5	475 mAh g^{-1} for the 200 cycles, 1 C.	[S14]
MNSs@d-Ti_3C_2/S	blank	3.7	474 mAh g^{-1} for 500 cycles, 1 C.	[S15]
KB/S@$Ti_3C_2T_x$	KB@ $Ti_3C_2T_x$	5.6	810 mAh g^{-1} (4.5 mAh cm^{-1})for the 1st cycle, 0.2 C, 600 mAh g^{-1} (3.4 mAh cm^{-1}) for 100 cycles, 0.2 C.	This work

Supplementary References

[S1] X. W. Fu, Y. Wang, L. S, W. H. Zhong, A polymeric nanocomposite interlayer as ion-transport-regulator for trapping polysulfides and stabilizing lithium metal. Energy Stor. Mater. **15**, 447-457 (2018). https://doi.org/10.1016/j.ensm.2018.06.025

[S2] Y. J. Li, P. Zhou, H. Li, T. T Gao, L. Zhou et al., A freestanding flexible single-atom cobalt-based multifunctional interlayer toward reversible and durable

lithium-sulfur batteries. Small Methods **4**(3), 1900701 (2020). https://doi.org/10.1002/smtd.201900701

[S3] P. Q. Guo, D. Q. Liu, Z. J. Liu, X. N. Shang, Q. M. Liu, D. Y. He, Dual functional MoS_2/graphene interlayer as an efficient polysulfide barrier for advanced lithium-sulfur batteries. Electrochim. Acta **256**, 28-36 (2017). https://doi.org/10.1016/j.electacta.2017.10.003

[S4] J. Balach, T. Jaumann, S. Mühlenhoff, J. Eckert, L. Giebeler, Enhanced polysulphide redox reaction using a RuO_2 nanoparticle-decorated mesoporous carbon as functional separator coating for advanced lithium–sulphur batteries. Chem. Comm. **52**(52), 8134-8137 (2016). https://doi.org/10.1039/C6CC03743G

[S5] F. Wu, S. Y. Zhao, L. Chen, Y. Lu, Y. F. Su et al., Metal-organic frameworks composites threaded on the CNT knitted separator for suppressing the shuttle effect of lithium sulfur batteries. Energy Stor. Mater, **14**, 383-391 (2018). https://doi.org/10.1016/j.ensm.2018.06.009

[S6] Y. Dong, S. Zheng, J. Qin, X. Zhao, H. Shi, X. Wang, Z. S. Wu, All-MXene-based integrated electrode constructed by Ti_3C_2 nanoribbon framework host and nanosheet interlayer for high-energy-density Li–S batteries. ACS Nano **12**(3), 2381-2388 (2018). https://doi.org/10.1021/acsnano.7b07672

[S7] J. Song, D. Su, X. Xie, X. Guo, W. Bao, G. Shao, G. Wang, Immobilizing polysulfides with mxene-functionalized separators for stable lithium–sulfur batteries. ACS Appl. Mater. Interfaces **8**(43), 29427-29433 (2016). https://doi.org/10.1021/acsami.6b09027

[S8] N. Li, Y. Xie, S. Peng, X. Xiong, K. Han, Ultra-lightweight $Ti_3C_2T_x$ MXene modified separator for Li–S batteries: Thickness regulation enabled polysulfide inhibition and lithium ion transportation. J. Energy Chem. **42**, 116-125 (2020). https://doi.org/10.1016/j.jechem.2019.06.014

[S9] C. Lin, W. Zhang, L. Wang, Z. Wang, W. Zhao, W. Duan, J. Jin, A few-layered Ti_3C_2 nanosheet/glass fiber composite separator as a lithium polysulphide reservoir for high-performance lithium–sulfur batteries. J. Mater. Chem. A **4**(16), 5993-5998 (2016).https://doi.org/10.1039/C5TA10307J

[S10] L. Yin, G. Xu, P. Nie, H. Dou, X. Zhang, MXene debris modified eggshell membrane as separator for high-performance lithium-sulfur batteries. Chem. Eng. J. **352**, 695-703 (2018). https://doi.org/10.1016/j.cej.2018.07.063

[S11] X. T. Gao, Y. Xie, X. D. Zhu, K. N. Sun, X. M. Xie, Y. T. Liu, B. Ding, Ultrathin MXene nanosheets decorated with TiO_2 quantum dots as an efficient sulfur host toward fast and stable Li–S batteries. Small **14**(41), 1802443 (2018).https://doi.org/10.1002/smll.201802443

[S12] L. Yin, G. Xu, P. Nie, H. Dou, X. Zhang, MXene debris modified eggshell

membrane as separator for high-performance lithium-sulfur batteries. Chem. Eng. J. **352**, 695-703 (2018).https://doi.org/10.1016/j.cej.2018.07.063

[S13] J. Song, D. Su, X. Xie, X. Guo, W. Bao, G. Shao, G. Wang, Immobilizing polysulfides with mxene-functionalized separators for stable lithium–sulfur batteries. ACS Appl. Mater. Interfaces **8**(43), 29427-29433 (2016). https://doi.org/10.1021/acsami.6b09027

[S14] H. Y. Zhou, Z. Y. Sui, K. A, L. W. Lin, H. Y. Wang, B. H. Han, Investigating the electrocatalysis of Ti_3C_2/carbon hybrid in polysulfide conversion of lithium–sulfur batteries. ACS Appl. Mater. Interfaces **25**, 547-554 (2020). https://doi.org/10.1021/acsami.9b23006

[S15] H. Zhang, Q. Qi, P. G. Zhang, W. Zheng, J. Chen et al., Self-assembled 3D MnO_2 nanosheets@delaminated-Ti_3C_2 aerogel as sulfur host for lithium–sulfur battery cathodes. ACS Appl. Energy Mater. **2**(1), 705-714 (2018). https://doi.org/10.1021/acsaem.8b01765
